# Prolonged QT Syndrome and Seizure Secondary to Alkaline Earth Metal Deficiency: A Case Report

**DOI:** 10.1155/2011/863029

**Published:** 2011-07-14

**Authors:** A. McKinney, B. C. Keegan

**Affiliations:** Department of Medicine, Erne Hospital, Enniskillen BT74 6Ay, UK

## Abstract

*Introduction*. Alkaline earth metal deficiency is recognized as a cause of both seizure and long QT syndrome. Their deficiency can have significant repercussions on the function of cells, tissues, and organs of the body. An understanding of the role of electrolytes allows an appreciation of the significance of depleted levels on cell function. *Case Report*. A 65-year-old lady was admitted with symptoms of chest discomfort, vomiting, increased stoma output, and dizziness. Two days following admission she suffered a tonic-clonic seizure. ECG review demonstrated a prolonged QTc interval, raising the possibility of an underlying Torsades de Pointes as the precipitant. This was attributed to electrolyte disturbance arising as a result of multiple aetiologies. *Discussion*. This paper highlights the multisystem effects of electrolyte disturbance, with emphasis upon its role in precipitating cardiac arrhythmia and neurological symptoms.

## 1. Case Report

A 65-year-old lady, with a past history of ischaemic heart disease, peripheral vascular disease, essential hypertension, peptic ulcer disease, and ischaemic colitis requiring bowel resection with construction of a colostomy, was admitted to the medical ward of a district general hospital with a 2-day history of chest pain, nausea, and vomiting, in conjunction with more longstanding “dizziness” and general malaise. At time of admission, her regular medications included clopidogrel, indapamide, nebivolol, and esomeprazole. She had no known allergies and no family history of note and was a nonsmoker and rarely consumed alcohol.

Initial examination revealed her to be clinically dehydrated, displaying a postural drop in blood pressure on standing. No other significant signs, however, were elicited. She was otherwise haemodynamically stable and apyrexial. Initial blood investigations returned demonstrating a mild hypokalaemia (K^+^3.2), hypocalcaemia (corrected Ca^2+^1.7), urea 7.4 mmol/l, creatinine 140 mmol/l, CRP 11, and a “random” troponin of 0.09 (normal range <0.03). Magnesium levels were not routinely measured on admission to the emergency department. Liver and thyroid function, fasting lipids and glucose, in addition to amylase and a short synacthen test were all normal.

**Figure 1:**
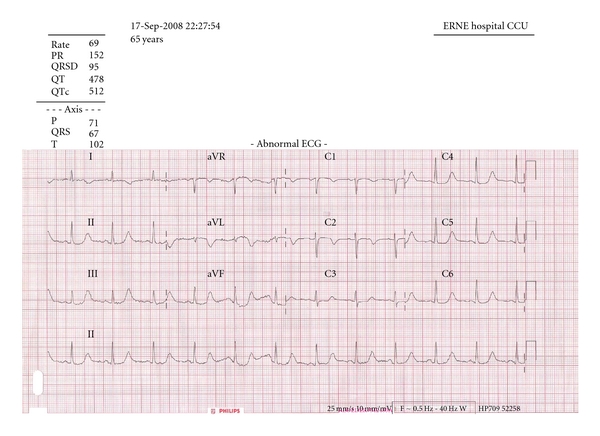
12-lead ECG displaying the patient's prolonged QT interval (QTc 512 milliseconds).

In view of the elevated troponin and in the context of chest pain, a 12-lead ECG was performed. This was documented as showing her to be in normal sinus rhythm with T-wave inversion in leads I and aVL, in addition to ST segment depression in lead V3 only (Figure 1). Despite the patient's impaired renal function being a potential contributor to the elevated troponin level, it was felt that there was sufficient evidence to justify commencing the patient on the “Acute Coronary Syndrome” treatment protocol. She was given a loading dose of aspirin and clopidogrel, as well as low-molecular-weight heparin. Other initial treatment included intravenous (IV) fluids, electrolyte replacement, and antiemetics to address her dehydration, electrolyte disturbance, and nausea, respectively. She was transferred to the coronary care unit for monitoring with follow-up blood tests including urea and electrolytes, bone profile, and troponin levels planned to assess the effect of the above interventions.

Following a consultant assessment on the ward round later that day, the above treatment measures were approved and continued. Her diagnoses were listed as follows:

1. possible acute coronary syndrome,
2. dehydration secondary to a likely viral gastroenteritis.

On review again the following morning, she appeared to feel better and blood tests had improved. Two days following admission, it was documented that output from her colostomy had increased and she remained on IV fluids. That afternoon she was the subject of a cardiac arrest call as she was witnessed to have collapsed on the ward. On arrival at her bedside the patient was found to be in a collapsed state with clonic movements of her limbs and clenched jaws. A nasopharyngeal airway was inserted, high flow oxygen applied, IV access obtained, and further blood samples including an arterial blood sample were taken. The patient was administered IV lorazepam and an ECG and portable chest X-ray were performed on resolution of seizure activity. The patient was transferred to the High Dependency Unit (HDU) where the investigations listed above were reviewed. 

## 2. Results

Her magnesium was low with levels of <0.27 mmol/l, in conjunction with a low calcium of 1.75 mmol/l corrected, and potassium was also on the lower side at 3.1 mmol/l. An arterial blood gas sample taken on 10 L/minute oxygen demonstrated a metabolic acidosis with high anion gap (pH 6.98, pO2 19.2 kPa, pCO2 6.4 kPa, base excess −20.3 mmol/l, Bicarbonate 11.3 mmol/l, and Lactate >15 mmol/l). Her chest X-ray was reported as normal. It was at this point, upon review of ECGs prolonged QTc intervals were noted.

A repeat ABG undertaken on admission to HDU when she had a Glasgow Coma Scale score of 15/15 found all values back within normal limits, indicating that the state of acidosis was transient. Further treatment instituted at this point included intravenous magnesium and calcium replacement in addition to potassium replacement. As electrolyte levels normalised, amiodarone was added on occurrence of new onset atrial fibrillation with rapid ventricular rate. 

It was concluded that vomiting and increased output from the colostomy amongst other causes such as drugs and intravenous fluids had resulted in depletion of the alkaline metals, magnesium, and calcium with subsequent prolongation of the patient's QT interval. As a result, she was felt to have collapsed and demonstrated seizure activity either as a result of arrhythmia such as Torsades de Pointes with collapse due to cerebral hypoperfusion secondary to the arrhythmia or directly due to seizure provoked by alkaline metal deficiency itself. It was felt that the profound metabolic acidosis was as a result of the collapse and transient anaerobic state. Unfortunately a copy of the patient's telemetry was not available because the unit had been removed as she had, despite a rapid deterioration, shown signs of improvement in the day prior to her collapse.

Over the next five days the patient's fluid and electrolyte balance was monitored, with gradual return towards normal and concurrent improvement of nausea and dizziness. Over the same period also, her stoma output was noted to reduce. QTc likewise improved, falling from 507 ms on the morning of event to 455 ms some days later. She was discharged home on oral electrolyte supplementation, with repeat ECG performed when all electrolytes were within normal range demonstrating a QTc of 432 ms. Indeed prior ECGs from 2003 and 2005 revealed QTc's of 435 and 432 milliseconds respectively. 

## 3. Discussion

The alkaline earth metals are a series of elements belonging to Group 2A of the periodic table. They include beryllium, magnesium, calcium, strontium, barium, and radium. The oxides of this group of metals produce basic alkaline solutions, giving rise to the term “alkaline earth metals.” 

Whereas some of these metals are essential for life, others although useful in the wider medical sphere are unnecessary for living organisms, for example, beryllium, barium, and radium. Beryllium possesses a direct corrosive effect on tissue, and when dusts containing the organism are inhaled, berylliosis may arise [1]. Radium's initial medical uses are being replaced by other elements such as ^60^Co these days [2]. Being incorporated into bone like calcium, its radioactivity leads to bone and bone marrow cancers in those heavily exposed. Barium in its insoluble sulphate form can be taken orally in radiological investigations, being completely eliminated from the digestive tract. Soluble barium compounds are poisonous, causing cardiac arrhythmias and paralysis probably due to the ability of this element to block potassium channels [3].

The case highlighted above involved deficiencies of magnesium and calcium, and these will now be discussed in turn. However, without blood results, the clinical signs of cation depletion are often nonspecific and may not be immediately attributed to this aetiology. In order to correct such a problem it is important for physicians to understand the causes of such depletion and appreciate potential clinical signs. 

## 4. Magnesium

Magnesium is primarily an intracellular cation, being the fourth most common in the body, and is recognised as an important coenzyme. Any process involving the transfer, storage, and utilization of energy within the body will involve magnesium. Magnesium is also necessary for the production of parathyroid hormone. Like calcium and phosphate, most magnesium is found in the reservoir of bone. Absorption of dietary magnesium mainly occurs in the ileum, with serum levels primarily regulated by the kidney. The normal plasma range is 0.7–0.95 mmol/l with 60% of this is in the ionised form. Incidence of hypomagnesaemia has been reported as 10–20% of hospital admissions and in up to 30–80% of alcoholics with the latter due to an osmotic diuretic effect of alcohol [4], a similar mechanism seen with hyperglycaemia in some diabetics. The main causes of magnesium deficiency are illustrated in Table 1. Our case would have been subject to gastrointestinal and drug (diuretic ± PPI) influence, with intravenous fluids potentially additionally contributing. 

Clinical manifestations of hypomagnesaemia are unusual at levels >0.5 mmol/L. Some are shown in Table 2. Magnesium deficiency is a recognised cause of arrhythmias including Torsades de Pointes. In this situation, magnesium sulphate, as an IV infusion is useful in arrhythmia termination [5]. With less severe degrees of hypomagnesemia, oral magnesium supplementation may be adequate. It is felt from the point of view of seizures that hypomagnesaemia may remove an inhibitory influence from the N-methyl-D-aspartate- (NMDA-) type glutamate receptors, so triggering neuronal depolarisation [6]. Seizures may also arise due to the cerebral hypoperfusion that may accompany arrhythmias [7]. 

## 5. Calcium

Calcium is the most common cation in the body, with up to 99% stored within the skeleton. Of the other 1% found in extracellular fluid, around half exists in free ionised form and under half bound to plasma proteins predominantly albumin. A small quantity is complexed with anions. Ionised calcium plays an important role in the physiological function of cells, for example, intracellular messaging, clotting of blood, cardiac automaticity, and muscular excitation-contraction in addition to neuronal conduction. Deficiency may manifest as abnormalities in these processes, with symptoms as in Table 4.

Normal range for calcium is 2.1–2.55 mmol/l, including ionised, protein bound, and complexed calcium. Plasma ionised calcium is dependant upon protein levels and pH. Calcium levels are also influenced by hormones such as parathyroid hormone (PTH), 1,25 dihydroxycholecalciferol, calcitonin, corticosteroids, thyroxine, and oestrogen. Other electrolytes play a role in calcium homeostasis. Illustrating this, magnesium is required for normal PTH function. Hypomagnesaemia can cause hypocalcaemia due to impaired synthesis and/or release of PTH and impaired peripheral action of this hormone that normally acts to mobilise calcium from the “bone reservoir” to ensure that plasma levels are kept within the normal range [5]. Our case would have been subject to this physiological process with dual deficiency of cations. 

Vitamin D is a fat-soluble vitamin that is ingested in the diet and produced by the body as a result of exposure to ultraviolet (UV) light. Vitamin D3 is converted to 25-hydroxycholecalciferol in the liver and subsequently to 1,25-dihydroxycholecalciferol by the kidneys. The major action of this substrate is to increase calcium and phosphate absorption from the intestine and to reduce the loss of these electrolytes in the urine [5]. In this paper we assume that increased output through the stoma impaired this physiological process. Reduced gastric acid production due to proton pump inhibitors may also result in reduced calcium absorption, with diuretics contributing to increased loss. Other potential causes of hypocalcaemia are illustrated in Table 3. 

## 6. The Arrhythmogenic Influence of Physiological Disturbance

Arrhythmic risk is promoted by electrolyte, acid-base, and fluid balance abnormalities, as well as cardiac ischaemia. Each of these may be applied to the patient in this paper. It is not uncommon to encounter metabolic derangements in hospitalized patients, and these can often be attributed to a reduced intake or increased losses. Fortunately a large proportion of these derangements are transient and easily correctable but there is the potential for life-threatening cardiac embarrassment even in the presence of a structurally normal heart. In the context of electrolyte disturbance, impaired cardiac function occurs as a result of arrhythmia and reduced myocardial contractility with resultant ischaemia. Arrhythmias occur because of reentry circuits and abnormal automaticity within myocardial foci. This causes inefficient myocardial function, and ectopic beats may herald more problematic heart rhythms.

**Figure 2:**
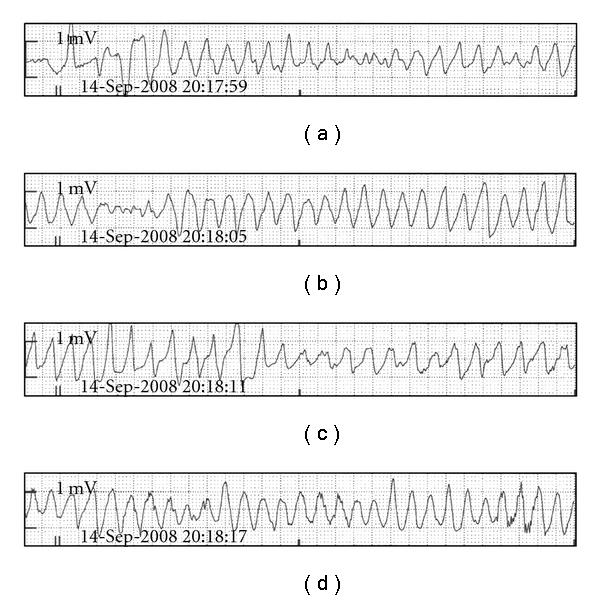
ECG appearance of Torsades de Pointes [10].

“After-depolarization” is a recognised mechanism through which electrolyte imbalance promotes arrhythmia. This can be further subdivided into “early” and “late” after-depolarization. Hypocalcaemia, hypokalaemia, hypomagnesaemia, and acidosis all promote this mechanism. It describes a low magnitude oscillation of the myocyte membrane potential during the period of repolarization. If this oscillation is large enough, it can stimulate ectopic activity by initiating further action potentials. This can progress to Torsades de Pointes, which is characterized by an undulation or twisting of the QRS axis during episodes of tachycardia (Figure 2). The generation of further action potentials will result in prolonged action of depolarizing ion channels with a resultant prolonged repolarization. This is recognised as a prolonged QT interval on the ECG [9].

The focus of this paper is “acquired” long QT syndrome. However, it is important to appreciate that the disorder is one of cardiac repolarizations caused by alterations in the transmembrane potassium and sodium currents. Congenital long QT syndrome is a disorder in processing these currents whereas acquired long QT syndrome is often as a result of an abnormality in the delivery of these substrates in order to initiate and propagate them.

The QT interval is calculated using Bazzett's formula (QTc = QT/√*R*-*R*), and this takes into account the effect of heart rate on the “uncorrected” QT interval. A prolonged QT interval is >0.44 seconds [7]. Acquired long QT syndrome is not always as a result of electrolyte disturbance. It may also be attributed to causes such as drugs, subarachnoid haemorrhage, myocardial ischaemia, protein sparing fasting, autonomic neuropathy, HIV, amongst others [11]. A drug-induced QTc increase from baseline is considered a clinically significant concern for risk of Torsade if more than 60 milliseconds [12].

It became clear that the aetiology of this patient's collapse was multifactorial in nature. It is likely that the patient had a history of preexisting electrolyte depletion secondary to intrinsic bowel pathology and diuretic therapy, coupled with insufficient dietary compensation. The increase in stoma output and resultant dehydration created an acute-on-chronic element to this presentation. Intravenous fluid therapy may also have created a dilutional effect on electrolyte concentrations, with proton pump inhibitors also recognised as being capable of inducing hypomagnesemia and hypocalcemia [13]. Although a magnesium level is not available from the set of blood tests taken on her initial presentation to the Emergency Department, followup at outpatient clinics has demonstrated that this patient's magnesium level has dropped to levels as low as 0.35 when her magnesium supplementation dose was reduced, and risen again when reinstated. 

## 7. Conclusion

If prolonged QT syndrome occurs as a result of a reversible cause this should be corrected to avoid cardiac embarrassment. In this paper our patient was successfully treated with fluid and electrolyte replacement. The importance of evaluating a collapsed patient's electrolyte balance, in addition to ECG monitoring, has been highlighted. If a patient is slow to respond to treatment consider a multifactorial cause for the patient's state. In retrospect, no aspect of this patient's care was particularly complex despite the diverse number of potential causes for her illness, though earlier appreciation of the presence of prolonged QTc may have resulted in swifter detection and correction of underlying electrolyte abnormalities. 

## Figures and Tables

**Table 1:** Causes of hypomagnesaemia.

Gastrointestinal disorders (e.g., diarrhoea, malabsorption, short-bowel syndrome, pancreatitis, and fistulae)
Alcohol dependence
Endocrine disorders (e.g., hyperparathyroidism, hyperthyroidism, diabetes, Conn's syndrome, and hyperaldosteronism)
Renal losses (e.g., renal tubular acidosis, postobstructive diuresis, and diuretic phase of acute tubular necrosis)
Drugs, for example, aminoglycosides, diuretics, penicillins, and proton pump inhibitors

**Table 2:** Clinical manifestations of hypomagnesemia.

Generalised weakness
Confusion, irritability, depression, or psychosis
Vertigo and ataxia
Tachyarrhythmia (Torsades de Pointes) and enhanced digoxin toxicity
Seizure activity
Biochemical disturbance (hypokalaemia, hypocalcaemia, and acidosis) [8]

**Table 3:** Causes of hypocalcemia.

Factitious (sample contamination with EDTA)
Normal plasma ionised with reduced total calcium (hypoalbuminaemia)
Reduced plasma ionised with normal total calcium (respiratory alkalosis, citrate toxicity found in transfusion)
Reduced plasma ionised and total calcium
(i) Decreased PTH activity (hypoparathyroidism, pseudohypoparathyroidism, and hypomagnesaemia)
(ii) Vitamin D deficiency
(iii) Excessive calcium losses (critical illness, malabsorption, pancreatitis, and diuresis)

**Table 4:** Clinical features of hypocalcemia.

Tetany (including laryngospasm)
Paraesthesia
Cramps
Mental changes
Areflexia
Seizures
Reduced cardiac output [6]

## References

[1] Meyer KC (1994). Beryllium and lung disease. *Chest*.

[2] http://www.webelements.com/radium/.

[3] Patnaik P (2003). *Handbook of Inorganic Chemical Compounds*.

[4] Wu C, Kenny MA (1996). Circulating total and ionized magnesium after ethanol ingestion. *Clinical Chemistry*.

[5] Baker SB, Worthley LIG (2002). The essentials of calcium, magnesium and phosphate metabolism: part I. Physiology. *Critical Care and Resuscitation*.

[6] Weisleder P, Tobin JA, Kerrigan JF, Bodensteiner JB (2002). Hypomagnesemic seizures: case report and presumed pathophysiology. *Journal of Child Neurology*.

[7] Hunt DPJ, Tang K (2005). Long QT syndrome presenting as epileptic seizures in an adult. *Emergency Medicine Journal*.

[8] Baker SB, Worthley LIG (2002). The essentials of calcium, magnesium and phosphate metabolism: part II. Disorders. *Critical Care and Resuscitation*.

[9] Cubbon RM, Kearney MT (2007). Acute metabolic derangement and the heart. *British Journal of Diabetes and Vascular Disease*.

[10] Chong DWS, Ankolekar SJ, McDonald J (2009). Sotalol induced QT prolongation and torsades de pointes. *British Medical Journal Case Reports*.

[11] Chiang CE (2004). Congenital and acquired long QT syndrome. Current concepts and management. *Cardiology in Review*.

[12] Owens RC (2004). QT prolongation with antimicrobial agents: understanding the significance. *Drugs*.

[13] Kuipers MT, Thang HD, Arntzenius AB (2009). Hypomagnesaemia due to use of proton pump inhibitors—a review. *Netherlands Journal of Medicine*.

